# Time Trends in Colorectal Cancer Incidence Rates by Income and Age at Diagnosis in Canada From 1992 to 2016

**DOI:** 10.1001/jamanetworkopen.2021.17556

**Published:** 2021-07-19

**Authors:** Kathleen M. Decker, Pascal Lambert, Jen Bravo, Alain Demers, Harminder Singh

**Affiliations:** 1 Department of Community Health Sciences, University of Manitoba, Winnipeg, Manitoba, Canada; 2 CancerCare Manitoba Research Institute, CancerCare Manitoba, Winnipeg, Manitoba, Canada; 3 Department of Epidemiology and Cancer Registry, CancerCare Manitoba, Winnipeg, Manitoba, Canada; 4 Public Health Agency of Canada, Ottawa, Ontario, Canada; 5 Department of Internal Medicine, University of Manitoba, Winnipeg, Manitoba, Canada

## Abstract

**Importance:**

Colorectal cancer (CRC) incidence rates among individuals younger than 50 years have been increasing in many countries, including Canada. Whether changes in CRC incidence rates over time are uniform across income and age groups remains unknown.

**Objective:**

To examine time trends in CRC incidence rates in Canada by area-level average household income by age group from 1992 to 2016.

**Design, Setting, and Participants:**

A population-based, retrospective cohort study was conducted including individuals aged 20 years or older diagnosed with CRC in Canada (excluding Québec) from 1992 to 2016. Data analysis was performed from February 27 to September 28, 2020.

**Exposures:**

Average household income was determined by linking an individual’s postal code at diagnosis from the Canadian Cancer Registry to Canadian Census data. Average household income was then categorized into quintiles from Q1, the lowest income group, to Q5, the highest income group.

**Main Outcomes and Measures:**

Canadian Cancer Registry data were used to determine CRC incidence rates from 1992 to 2016.

**Results:**

There were 340 790 cases of CRC diagnosed from 1992 to 2016; 11 790 cases (3.5%) were diagnosed in individuals aged 20 to 44 years. Colorectal cancer incidence rates for individuals aged 20 to 44 years increased for all income quintiles, with higher incidence rates in the lower income quintiles. The ratio between the maximum and minimum CRC incidence rates was highest among the 20- to 29-year age group from 1992 to 1996 (ratio, 2.67; 95% CI, 1.47-4.83) and 2012 to 2016 (ratio, 2.00; 95% CI, 1.29-3.10). For individuals aged 45 to 49 years, CRC incidence rates increased only for individuals in the lower income quintiles (eg, incidence in Q1, 1992-1996 vs 2012-2016: 4.54 per 100 000 [95% CI, 4.05-5.03] vs 5.37 per 100 000 [95% CI, 4.91-5.83]), with higher incidence rates in the high income quintiles (eg, incidence rate for Q5 in 1992-1996: 5.92 per 100 000 [95% CI, 5.36-6.48]). For those aged 50 to 54 years, CRC incidence rates were stable for all income quintiles, with less variability between income quintiles. For individuals aged 55 to 74 years, CRC incidence rates were stable or decreased for all income quintiles (eg, incidence rate for age 55-59 years in Q5, 1992-1992 vs 2012-2016: 17.97 per 100 000 [95% CI, 16.76-19.18] vs 14.56 per 100 000 [95% CI, 13.80-15.32]), and there was less variability in the rates by income quintile, particularly from 2012 to 2016. After age 75 years, CRC incidence rates were stable or decreased (eg, incidence rate for age 75-79 years, 1992-1996 vs 2012-2016: 66.43 per 100 000 [65.00-67.87] vs 57.34 per 100 000 [56.24-58.45]), were highest for the lower income quintiles, and variability between income quintiles increased relative to younger age groups.

**Conclusions and Relevance:**

In this cohort study, trends in CRC incidence rates in Canada differed by age group and income quintile. These results suggest that, although population-based screening can reduce income disparities, targeted interventions and further research are needed to address the increasing CRC incidence rate among younger individuals in Canada, particularly in the lower income quintiles.

## Key Points

**Question:**

Do time trends in colorectal cancer incidence rates by age group in Canada differ by income quintile?

**Findings:**

In this cohort study of 340 790 individuals aged 20 to 44 years, colorectal cancer incidence rates increased and were higher in the lower income quintiles. Colorectal cancer incidence rates were stable or decreased among individuals aged 45 to 74 years, with little variability between income quintiles, and decreased for individuals aged 75 years or older, with higher rates in the lower income quintiles.

**Meaning:**

The findings of this study suggest that targeted interventions and further research are needed to address the increasing colorectal cancer incidence rates among younger individuals in Canada, particularly in the lower income quintiles.

## Teaser

This cohort study examines changes in the incidence of colorectal cancer from 1992 to 2016 in individuals in Canada by age group and income quintile.

## Introduction

Colorectal cancer (CRC) incidence rates for all ages combined have been decreasing in North America, albeit much slower and starting in later years in Canada than in the US.^1,2^ However, studies from multiple countries, including Canada, have found that CRC incidence rates are increasing in individuals younger than 50 years, particularly for rectal cancer.^2,3,4,5,6,7,8,9^ An association between CRC incidence and birth cohort has been reported, with more recent younger Canadian cohorts at greater risk than those born earlier.^8^ Although the reasons for the increase are not yet known, the increased use of colonoscopy and flexible sigmoidoscopy for diagnostic purposes cannot fully explain the increased number of cases, and it is likely that lifestyle factors, such as obesity, diet, and exercise, play an important role.^2,10,11^ Many of these lifestyle factors for CRC are substantially different among varying socioeconomic groups.^12^ Individuals with lower socioeconomic status (SES) may have higher CRC incidence rates than those with higher SES, although the pattern differs by colon site and country of residence.^13^ However, there are limited data on an association between SES and CRC in the younger population and, to our knowledge, Canadian or US studies have not assessed the association between SES and CRC incidence rates in younger age groups.^8^ Therefore, it remains unknown whether the increased CRC incidence rates among the younger population differ by income subgroups. The objective of this study was to examine time trends in CRC incidence rates in Canada by average household income among different age groups between 1992 and 2016.

## Methods

### Study Design and Population

This study was a population-based retrospective cohort study. Individuals aged 20 years or older diagnosed with CRC in Canada from 1992 to 2016 were included. The province of Québec was excluded because information was only available until 2010. The following *International Classification of Diseases for Oncology (Third Edition)* codes from 1992 onward were included: C18.0 (cecum), C18.2 to C18.9 (ascending colon, hepatic flexure, transverse colon, splenic flexure, descending colon, sigmoid colon, and large intestine not otherwise specified), C19.9 (rectosigmoid junction), C20.9 (rectum), and C26.0 (malignant neoplasm of intestinal tract, part unspecified).^14^ These codes are used by Canadian Cancer Statistics publications to define CRC. The study was approved by the University of Manitoba’s Health Research Ethics Board and the Research and Resource Impact Committee at CancerCare Manitoba. Written informed consent was waived because all data were deidentified. This study followed the Strengthening the Reporting of Observational Studies in Epidemiology (STROBE) reporting guideline.

### Data Sources

We used data from the Canadian Cancer Registry to determine the CRC incidence in Canada from 1992 (the year the Canadian Cancer Registry was created) to 2016. The Canadian Cancer Registry is a person-level database that includes clinical (tumor location, histologic subtype, and grade) and demographic (including 6-digit postal code) information about Canadian residents diagnosed with new cases of cancer.^1^ Because the Canadian Cancer Registry does not contain information about SES, area-level average household income was determined by linking postal code at diagnosis from the Canadian Cancer Registry to Statistics Canada’s Postal Code Conversion File to identify the Canadian Census dissemination area in which an individual resided. To account for changes in area-level income over time, the Postal Code Conversion File version that corresponded to an individual’s year of diagnosis was used. Postal code is complete for 98% of cases in the Canadian Cancer Registry.^15^ An individual’s dissemination area was then linked to Canadian population census data closest to the year of CRC diagnosis to determine average household income at the time of diagnosis. Average household income was categorized into quintiles from Q1 (lowest) to Q5 (highest).

### Statistical Analysis

Colorectal cancer incidence rates and 95% CIs were calculated by age group (20-29, 30-39, 40-44, 45-49, 50-54, 55-59, 60-64, 65-59, 70-74, 75-79, 80-84, 85-89, and ≥90 years) and income quintile (Q1, Q2, Q3, Q4, and Q5) for five 5-year periods (1992-1996, 1997-2001, 2002-2006, 2007-2011, and 2012-2016). The average percentage change (APC) over each 5-year period by age and income was calculated using the Joinpoint Regression program, version 4.8.0.1 (developed by Surveillance Epidemiology and End Results, National Cancer Institute). Two-tailed, unpaired analyses with differences at *P* < .05 represented a significant change in the APC over the 5-year period. Variability in rates by income quintile was determined by calculating the ratio between the maximum and minimum incidence rates within each age group. Therefore, if the ratio between the maximum and the minimum incidence rate was 1, there was no variability between the income quintiles. As the ratio became greater than 1, there was more variability between the income quintiles. The 95% CIs were calculated using Poisson regression. We also calculated the ratio of CRC incidence rates by SES groups over time, with the highest income quintile (Q5) as the reference group. *P* values for the ratios were calculated using the Wald test from the Poisson regression. To ensure confidentiality, all counts were randomly rounded to a lower or higher multiple of 5. Data analysis was performed from February 27 to September 28, 2020. Analyses were conducted using R, version 4.0.4 (R Project for Statistical Computing) and SAS, version 9.4 (SAS Institute Inc).

## Results

**Figure 1.**
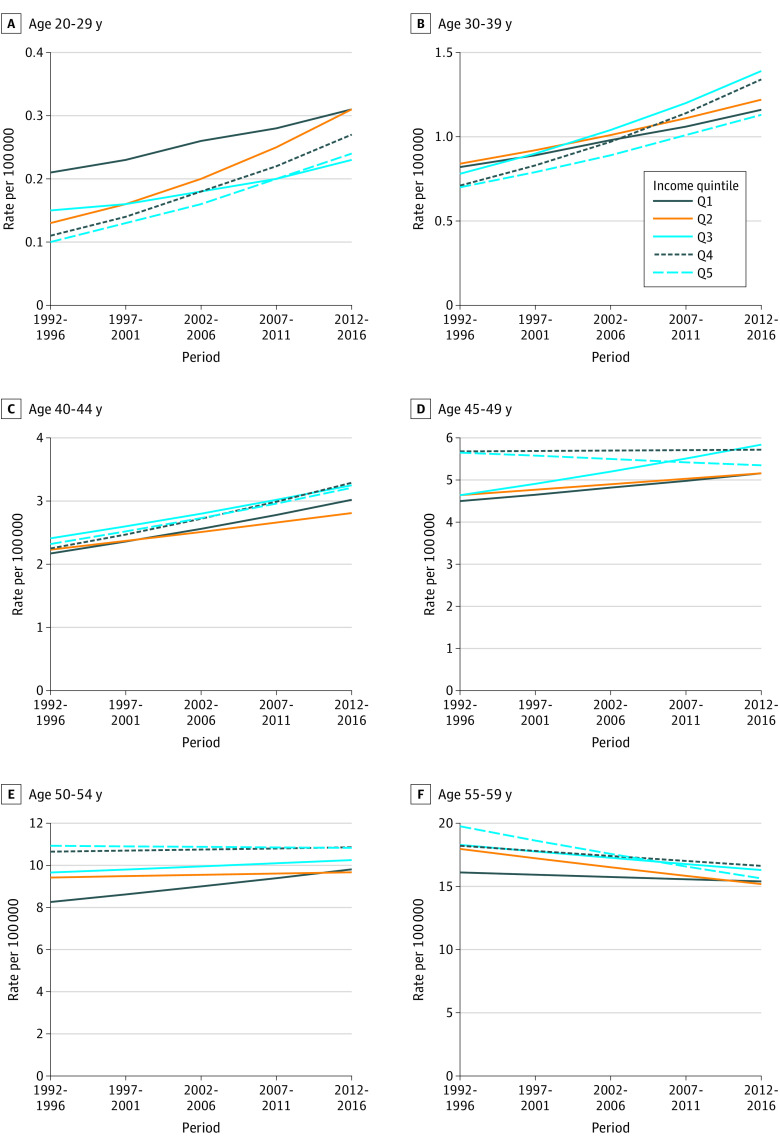
Time Trends in Colorectal Cancer Incidence Rate per 100 000 Individuals by Income Quintile and Age Group for Both Sexes Combined in Canada by 5-Year Time Periods From 1992 to 2016

There were 340 790 cases of CRC diagnosed in Canadian residents from 1992 to 2016 (Table 1). A total of 11 790 CRC cases (3.5%) were diagnosed in individuals aged 20 to 44 years. A slightly higher percentage of cases was diagnosed among the lowest and middle income quintiles (Q1, 20.5%; Q2, 21.3%; and Q3, 20.0%) compared with the highest income quintiles (Q4, 19.4%; Q5, 19.0%). Figure 1 and eFigure 1 in the Supplement illustrate the time trend in Joinpoint-predicted CRC incidence rate per 100 000 by income quintile and age group for both sexes combined in Canada by five 5-year periods from 1992-1996 to 2012-2016. Colorectal cancer incidence rates increased over time for individuals aged 20 to 29 years in all income quintiles from 0.14 per 100 000 (95% CI, 0.11-0.17) in 1992-1996 to 0.25 per 100 000 (95% CI, 0.22-0.29) in 2012-2016. Colorectal cancer incidence rates were highest for the lowest income quintile (Q1) and lowest for the highest income quintile (Q5) across all periods. The increase was significant for Q2 (APC, 24.7%; 95% CI, 10.8%-40.3%), Q4 (APC, 23.7%; 95% CI, 13.9%-34.3%), and Q5 (APC, 23.8%; 95% CI, 7.0%-43.3%) (Table 2). Colorectal cancer incidence rates also increased for individuals aged 30 to 39 years in all income quintiles from 0.79 per 100 000 (95% CI, 0.73-0.85) in 1992-1996 to 1.29 (95% CI, 1.22-1.36) in 2012-2016. Colorectal cancer incidence rates were highest for Q2 from 1992-1996 to 2002-2006, after which the incidence rates were highest for the middle income quintile (Q3). The increase was significant for Q2 (APC, 9.8%; 95% CI, 2.5%-17.5%), Q3 (APC, 15.5%; 95% CI, 9.7%-21.7%), Q4 (APC, 17.1%; 95% CI, 12.4%-22.1%), and Q5 (APC, 12.7%; 95% CI, 2.2%-24.3%). Colorectal cancer incidence rates increased for all individuals aged 40 to 44 years from 2.41 per 100 000 (95% CI, 2.26-2.55) in 1992-1996 to 3.30 per 100 000 (95% CI, 3.13-3.46) in 2012-2016 and was highest for Q3 until 2007-2011, after which the CRC incidence rate was the highest for Q4. The increase was significant for Q1 (APC, 8.6%; 95% CI, 4.5%-12.9%) and Q5 (APC, 8.4%; 95% CI, 3.2%-14.0%). For individuals aged 45 to 49 years, CRC incidence rates increased from 1992-1996 to 2012-2016 in the lowest income quintiles: Q1, 4.54 per 100 000 (95% CI, 4.05-5.03) to 5.37 per 100 000 (95% CI, 4.91-5.83); Q2, 5.16 per 100 000 (95% CI, 4.64-5.68) to 5.42 per 100 000 (95% CI, 4.56-5.88); and Q3, 4.40 per 100 000 (95% CI, 3.92-4.89) to 5.83 per 100 000 (95% CI, 5.35-6.31). The incidence was stable for Q4 (ie, no significant increase or decrease) and decreased for Q5 (5.92 per 100 000; 95% CI, 5.36-6.48 to 5.68; 95% CI, 5.20-6.15). Colorectal cancer incidence rates were highest for the highest income quintiles (Q4 and Q5). The only significant change was an increase for Q3 (APC, 6.0%; 95% CI, 0.1%-12.1%).

**Table 1.** Colorectal Cancer Incidence Rate per 100 000 by Age Group and Income Quintile, Canada, 1992 to 2016^a^

Age, y	Income quintile^b^	Cases. No.	1992-1996		1997-2001		2002-2006		2007-2011		2012-2016
			Rate per 100 000 (95% CI)	Cases, No.	Rate per 100 000 (95% CI)	Cases, No.	Rate per 100 000 (95% CI)	Cases, No.	Rate per 100 000 (95% CI)	Cases, No.	Rate per 100 000 (95% CI)
20-29	No.	880									
	All quintiles	115	0.14 (0.11-0.17)	125	0.16 (0.13-0.19)	175	0.21 (0.18-0.24)	225	0.25 (0.22-0.28)	240	0.25 (0.22-0.29)
	Q1	40	0.24 (0.17-0.33)	30	0.19 (0.13-0.27)	40	0.24 (0.17-0.33)	55	0.31 (0.23-0.40)	60	0.32 (0.24-0.41)
	Q2	20	0.12 (0.07-0.19)	25	0.16 (0.10-0.23)	40	0.24 (0.17-0.33)	45	0.25 (0.18-0.34)	55	0.29 (0.22-0.38)
	Q3	20	0.12 (0.07-0.19)	25	0.16 (0.10-0.23)	40	0.24 (0.17-0.33)	50	0.28 (0.21-0.37)	30	0.16 (0.11-0.23)
	Q4	20	0.12 (0.07-0.19)	20	0.13 (0.08-0.19)	30	0.18 (0.12-0.26)	40	0.22 (0.16-0.30)	50	0.27 (0.20-0.35)
	Q5	15	0.09 (0.05-0.15)	25	0.16 (0.10-0.23)	25	0.15 (0.10-0.22)	35	0.20 (0.14-0.27)	45	0.24 (0.17-0.32)
30-39	No.	4570									
	All	760	0.79 (0.73-0.85)	840	0.88 (0.82-0.94)	820	0.92 (0.85-0.98)	965	1.10 (1.03-1.17)	1185	1.29 (1.22-1.36)
	Q1	155	0.81 (0.68-0.93)	185	0.97 (0.83-1.11)	155	0.87 (0.73-1.00)	200	1.14 (0.98-1.30)	210	1.14 (0.99-1.30)
	Q2	170	0.89 (0.75-1.02)	175	0.92 (0.78-1.05)	165	0.92 (0.78-1.06)	195	1.11 (0.96-1.27)	235	1.28 (1.11-1.44)
	Q3	150	0.78 (0.66-0.91)	180	0.94 (0.80-1.08)	175	0.98 (0.83-1.12)	205	1.17 (1.01-1.33)	265	1.44 (1.27-1.61)
	Q4	140	0.73 (0.61-0.85)	150	0.79 (0.66-0.91)	180	1.00 (0.86-1.15)	200	1.14 (0.98-1.30)	245	1.33 (1.16-1.50)
	Q5	145	0.76 (0.63-0.88)	150	0.79 (0.66-0.91)	145	0.81 (0.68-0.94)	165	0.94 (0.80-1.09)	230	1.25 (1.09-1.41)
40-44	No.	6340									
	All	1005	2.41 (2.26-2.55)	1180	2.46 (2.32-2.60)	1255	2.41 (2.28-2.55)	1380	2.87 (2.72-3.02)	1520	3.30 (3.13-3.46)
	Q1	185	2.21 (1.90-2.53)	225	2.35 (2.04-2.65)	265	2.55 (2.24-2.85)	255	2.65 (2.33-2.98)	290	3.15 (2.78-3.51)
	Q2	195	2.33 (2.01-2.66)	245	2.56 (2.24-2.88)	220	2.11 (1.83-2.39)	240	2.50 (2.18-2.81)	290	3.15 (2.78-3.51)
	Q3	215	2.57 (2.23-2.92)	245	2.56 (2.24-2.88)	270	2.59 (2.28-2.90)	275	2.86 (2.52-3.20)	325	3.53 (3.14-3.91)
	Q4	205	2.45 (2.12-2.79)	235	2.45 (2.14-2.76)	225	2.16 (1.88-2.44)	325	3.38 (3.01-3.75)	310	3.36 (2.99-3.74)
	Q5	205	2.45 (2.12-2.79)	230	2.40 (2.09-2.71)	275	2.64 (2.33-2.95)	285	2.97 (2.62-3.31)	305	3.31 (2.94-3.68)
45-49	No.	11 985									
	All	1865	5.13 (4.9-5.37)	2165	5.12 (4.91-5.34)	2470	5.09 (4.89-5.29)	2740	5.21 (5.01-5.40)	2745	5.62 (5.41-5.83)
	Q1	330	4.54 (4.05-5.03)	415	4.91 (4.44-5.38)	425	4.38 (3.96-4.80)	520	4.94 (4.52-5.37)	525	5.37 (4.91-5.83)
	Q2	375	5.16 (4.64-5.68)	360	4.26 (3.82-4.70)	455	4.69 (4.26-5.12)	530	5.04 (4.61-5.46)	530	5.42 (4.56-5.88)
	Q3	320	4.40 (3.92-4.89)	450	5.32 (4.83-5.82)	500	5.15 (4.70-5.61)	570	5.42 (4.97-5.86)	570	5.83 (5.35-6.31)
	Q4	410	5.64 (5.10-6.19)	485	5.74 (5.23-6.25)	560	5.77 (5.29-6.25)	585	5.56 (5.11-6.01)	565	5.78 (5.30-6.26)
	Q5	430	5.92 (5.36-6.48)	455	5.38 (4.89-5.88)	530	5.46 (5.00-6.25)	535	5.08 (4.65-5.51)	555	5.68 (5.20-6.15)
50-54	No.	20 945									
	All	2745	9.88 (9.51-10.25)	3530	9.73 (9.41-10.05)	4265	10.08 (9.78-10.38)	4975	10.20 (9.91-10.48)	5430	10.28 (10.00-10.55)
	Q1	485	8.73 (7.95-9.51)	580	7.99 (7.34-8.65)	755	8.92 (8.28-9.56)	935	9.58 (8.97-10.19)	1045	9.89 (9.29-10.49)
	Q2	535	9.63 (8.81-10.45)	660	9.10 (8.40-9.79)	830	9.81 (9.14-10.47)	925	9.48 (8.87-10.09)	1030	9.75 (9.15-10.34)
	Q3	540	9.72 (8.90-10.54)	715	9.85 (9.13-10.58)	810	9.57 (8.91-10.23)	1020	10.45 (9.81-11.09)	1075	10.17 (9.56-10.78)
	Q4	595	10.71 (9.85-11.57)	760	10.47 (9.73-11.22)	940	11.11 (10.40-11.82)	1030	10.55 (9.91-11.20)	1155	10.93 (10.30-11.56)
	Q5	590	10.62 (9.76-11.48)	815	11.23 (10.46-12.00)	930	10.99 (10.28-11.69)	1065	10.91 (10.26-11.57)	1125	10.65 (10.02-11.27)
55-59	No.	29 655									
	All	4170	17.73 (17.20-18.27)	4825	17.55 (17.06-18.05)	6200	17.26 (16.83-17.69)	6985	16.69 (16.29-17.08)	7475	15.44 (15.09-15.79)
	Q1	780	16.59 (15.42-17.75)	850	15.46 (14.42-16.50)	1130	15.73 (14.81-16.64)	1265	15.11 (14.28-15.94)	1535	15.85 (15.06-16.64)
	Q2	860	18.29 (17.06-19.51)	935	17.01 (15.92-18.10)	1185	16.49 (15.55-17.43)	1285	15.35 (14.51-16.19)	1510	15.59 (14.81-16.38)
	Q3	850	18.08 (16.86-19.29)	975	17.74 (16.62-18.85)	1240	17.26 (16.30-18.22)	1475	17.62 (16.72-18.52)	1520	15.70 (14.91-16.49)
	Q4	835	17.76 (16.55-18.96)	970	17.64 (16.53-18.75)	1280	17.81 (16.84-18.79)	1545	18.45 (17.53-19.37)	1500	15.49 (14.71-16.27)
	Q5	845	17.97 (16.76-19.18)	1095	19.92 (18.74-21.10)	1365	19.00 (17.99-20.00)	1415	16.90 (16.02-17.78)	1410	14.56 (13.80-15.32)
60-64	No.	38 640									
	All	6130	27.51 (26.82-28.20)	6425	27.91 (27.23-28.59)	7365	27.22 (26.60-27.84)	9135	25.88 (25.35-26.41)	9585	23.31 (22.84-23.77)
	Q1	1175	26.36 (24.85-27.87)	1195	25.96 (24.48-27.43)	1395	25.78 (24.43-27.13)	1700	24.08 (22.94-25.23)	2000	24.31 (23.25-25.38)
	Q2	1220	27.37 (25.84-28.91)	1355	29.43 (27.86-31.00)	1505	27.81 (26.41-29.22)	1835	26.00 (24.81-27.18)	1950	23.71 (22.65-24.76)
	Q3	1260	28.27 (26.71-29.83)	1295	28.13 (26.60-29.66)	1465	27.07 (25.69-28.46)	1810	25.64 (24.46-26.82)	1905	23.16 (22.12-24.20)
	Q4	1255	28.16 (26.60-29.71)	1290	28.02 (26.49-29.55)	1475	27.26 (25.87-28.65)	1885	26.70 (25.50-27.91)	1890	22.98 (21.94-24.01)
	Q5	1220	27.37 (25.84-28.91)	1290	28.02 (26.49-29.55)	1525	28.18 (26.77-29.60)	1905	26.99 (25.77-28.20)	1840	22.37 (21.35-23.39)
65-69	No.	47 320									
	All	8235	40.04 (39.17-40.90)	8555	40.31 (39.45-41.16)	8980	40.76 (39.92-41.60)	10 055	38.69 (37.94-39.45)	11 495	33.80 (33.19-34.42)
	Q1	1700	41.32 (39.36-43.29)	1730	40.76 (38.84-42.68)	1775	40.28 (38.41-42.16)	1970	37.91 (36.23-39.58)	2245	33.01 (31.64-34.38)
	Q2	1755	42.66 (40.66-44.65)	1795	42.29 (40.33-44.24)	1880	42.67 (40.74-44.59)	2000	38.48 (36.80-40.17)	2435	35.80 (34.38-37.23)
	Q3	1730	42.05 (40.07-44.03)	1715	40.40 (38.49-42.32)	1820	41.30 (39.41-43.20)	2140	41.18 (39.43-42.92)	2360	34.70 (33.30-36.10)
	Q4	1510	36.70 (34.85-38.55)	1700	40.05 (38.15-41.95)	1820	41.30 (39.41-43.20)	2025	38.96 (37.27-40.66)	2250	33.08 (31.72-34.45)
	Q5	1540	37.43 (35.56-39.30)	1615	38.05 (36.19-39.90)	1685	38.24 (36.41-40.07)	1920	36.94 (35.29-38.60)	2205	32.42 (31.07-33.77)
70-74	No.	52 160									
	All	9090	51.68 (50.62-52.74)	10 155	54.46 (53.40-55.52)	10 630	54.63 (53.59-55.67)	10 905	53.23 (52.23-54.23)	11 380	46.97 (46.11-47.84)
	Q1	1905	54.15 (51.72-56.58)	2085	55.91 (53.51-58.31)	2180	56.01 (53.66-58.36)	2170	52.97 (50.74-55.19)	2350	48.50 (46.54-50.46)
	Q2	1995	56.71 (54.22-59.20)	2160	57.92 (55.47-60.36)	2295	58.97 (56.56-61.38)	2325	56.75 (54.44-59.05)	2405	49.63 (47.65-51.62)
	Q3	1845	52.45 (50.05-54.84)	2085	55.91 (53.51-58.31)	2120	54.47 (52.15-56.79)	2230	54.43 (52.17-56.69)	2265	46.75 (44.82-48.67)
	Q4	1690	48.04 (45.75-50.33)	1950	52.29 (49.97-54.61)	2070	53.19 (50.90-55.48)	2115	51.62 (49.42-53.82)	2210	45.61 (43.71-47.51)
	Q5	1655	47.05 (44.78-49.31)	1875	50.28 (48.00-52.55)	1965	50.49 (48.26-52.72)	2065	50.40 (48.23-52.58)	2150	44.37 (42.50-46.25)
75-79	No.	50 630									
	All	8245	66.43 (65.00-67.87)	10 125	68.27 (66.94-69.60)	10 835	67.42 (66.16-68.69)	11 020	64.95 (63.74-66.16)	10 405	57.34 (56.24-58.45)
	Q1	1820	73.32 (69.95-76.69)	2175	73.33 (70.24-76.41)	2275	70.79 (67.88-73.69)	2245	66.16 (63.42-68.90)	2205	60.76 (58.23-63.30)
	Q2	1850	74.53 (71.13-77.92)	2235	75.35 (72.23-78.47)	2395	74.52 (71.53-77.50)	2420	71.32 (68.47-74.16)	2330	64.21 (61.60-66.81)
	Q3	1595	64.26 (61.10-67.41)	2055	69.28 (66.29-72.28)	2180	67.83 (64.98-70.68)	2250	66.31 (63.57-69.05)	2075	57.18 (54.72-59.64)
	Q4	1520	61.24 (58.16-64.31)	1830	61.70 (58.87-64.52)	1990	61.92 (59.20-64.64)	2045	60.27 (57.65-62.88)	1975	54.42 (52.02-56.82)
	Q5	1460	58.82 (55.80-61.83)	1830	61.70 (58.87-64.52)	1995	62.07 (59.35-64.80)	2060	60.71 (58.08-63.33)	1820	50.15 (47.85-52.46)
80-84	No.	41 565									
	All	6245	75.83 (73.95-77.71)	7365	79.04 (77.24-80.85)	8820	76.23 (74.64-77.82)	9490	74.78 (73.27-76.28)	9645	70.57 (69.17-71.98)
	Q1	1485	90.16 (85.57-94.74)	1720	92.30 (87.94-96.66)	1940	83.83 (80.10-87.56)	1965	77.42 (73.99-80.84)	2130	77.93 (74.62-81.24)
	Q2	1400	85.00 (80.54-89.45)	1655	88.81 (84.53-93.09)	1970	85.13 (81.37-88.89)	2080	81.95 (78.42-85.47)	2100	76.83 (73.54-80.12)
	Q3	1250	75.89 (71.68-80.09)	1445	77.54 (73.54-81.54)	1715	74.11 (70.60-77.62)	1895	74.66 (71.30-78.02)	1955	71.53 (68.35-74.70)
	Q4	1035	62.84 (59.01-66.66)	1260	67.61 (63.88-71.35)	1645	71.08 (67.65-74.52)	1795	70.72 (67.45-73.99)	1770	64.76 (61.74-67.77)
	Q5	1075	65.26 (61.36-69.16)	1285	68.95 (65.18-72.72)	1550	66.98 (63.64-70.31)	1755	69.14 (65.91-72.38)	1690	61.83 (58.88-64.78)
85-89	No.	25 220									
	All	3170	76.54 (73.87-79.20)	4280	84.31 (81.79-86.84)	5070	84.37 (82.05-86.70)	6335	82.60 (80.57-84.64)	6365	73.71 (71.90-75.52)
	Q1	765	92.35 (85.81-98.90)	1020	100.47 (94.30-106.64)	1185	98.60 (92.99-104.22)	1325	86.39 (81.73-91.04)	1495	86.57 (82.18-90.95)
	Q2	705	85.11 (78.83-91.39)	1000	98.50 (92.39-104.60)	1095	91.11 (85.72-96.51)	1425	92.91 (88.08-97.73)	1395	80.78 (76.53-85.01)
	Q3	590	71.23 (65.48-76.97)	795	78.31 (72.86-83.75)	975	81.13 (76.04-86.22)	1230	80.19 (75.71-84.67)	1230	71.22 (67.24-75.20)
	Q4	555	67.00 (61.43-72.57)	735	72.40 (67.16-77.63)	880	73.22 (68.38-78.06)	1175	76.61 (72.23-80.99)	1170	67.75 (63.87-71.63)
	Q5	555	67.00 (61.43-72.57)	730	71.90 (66.67-77.12)	935	77.80 (72.81-82.79)	1180	76.93 (72.54-81.32)	1075	62.25 (58.53-65.97)
≥90	No.	10 880									
	All	1305	62.97 (59.55-66.39)	1770	73.34 (69.92-76.76)	2225	72.97 (69.94-76.00)	2775	72.32 (69.63-75.02)	3140	61.78 (59.62-63.95)
	Q1	335	80.83 (72.17-89.48)	440	91.16 (82.64-99.67)	560	91.83 (84.22-99.43)	640	83.40 (76.94-89.86)	760	74.77 (69.46-80.09)
	Q2	290	69.97 (61.91-78.02)	420	87.01 (78.69-95.33)	460	75.43 (68.54-82.32)	600	78.19 (71.93-84.44)	690	67.89 (62.82-72.95)
	Q3	235	56.70 (49.45-63.95)	345	71.47 (63.93-79.02)	425	69.69 (63.06-76.32)	535	69.72 (63.81-75.63)	640	62.97 (58.09-67.84)
	Q4	230	55.49 (48.32-62.66)	265	54.90 (48.28-61.51)	395	64.77 (58.38-71.16)	520	67.76 (61.94-73.59)	545	53.62 (49.12-58.12)
	Q5	215	51.87 (44.94-58.81)	300	62.15 (55.12-69.19)	385	63.13 (56.82-69.44)	480	62.55 (56.95-68.15)	505	49.68 (45.35-54.02)

^a^ Total number of cases, 340 790. Sums vary because all numbers were rounded per Statistics Canada requirements.

^b^ Q1 income level represents the lowest income quintile; Q5 represents the highest income quintile.

**Table 2.** APC in Colorectal Cancer Incidence Rate by Age Group and Income Quintile, Canada, 1992 to 2016

Age, y	Income quintile^a^											
	Q1		Q2		Q3		Q4		Q5		All	
	APC (95% CI)	*P* value	APC (95% CI)	*P* value	APC (95% CI)	*P* value	APC (95% CI)	*P* value	APC (95% CI)	*P* value	APC (95% CI)	*P* value
20-29	10.7 (−5.6 to 29.9)	.14	24.7 (10.8 to 40.3)	.01	11.6 (−20.5 to 56.7)	.38	23.7 (13.9 to 34.3)	.004	23.8 (7.0 to 43.3)	.02	18.0 (8.7 to 28.2)	.008
30-39	9.0 (−0.8 to 19.8)	.06	9.8 (2.5 to 17.5)	.02	15.5 (9.7 to 21.7)	.003	17.1 (12.4 to 22.1)	.001	12.7 (2.2 to 24.3)	.03	12.8 (7.6 to 18.2)	.004
40-44	8.6 (4.5 to 12.9)	.006	5.9 (−7.4 to 21.1)	.27	7.7 (−0.7 to 16.9)	.06	10.0 (−6.3 to 29.1)	.16	8.4 (3.2 to 14.0)	.01	8.2 (0.5 to 16.4)	.04
45-49	3.5 (−3.3 to 10.7)	.21	2.7 (−6.8 to 13.2)	.45	6.0 (0.1 to 12.1)	.05	0.2 (−1.8 to 2.1)	.81	−1.4 (−7.2 to 4.8)	.52	2.0 (−1.1 to 5.1)	.13
50-54	4.4 (−1.3 to 10.4)	.09	0.7 (−2.6 to 4.0)	.57	1.5 (−1.6 to 4.7)	.22	0.5 (−2.2 to 3.2)	.61	−0.2 (−2.9 to 2.5)	.79	1.3 (0 to 2.5)	.05
55-59	−1.1 (−4.5 to 2.3)	.37	−4.1 (−6.7 to −1.5)	.02	−2.8 (−6.3 to 0.8)	.09	−2.3 (−8.5 to 4.4)	.35	−5.7 (−13.9 to 3.4)	.14	−3.2 (−5.6 to −0.8)	.02
60-64	−2.3 (−4.2 to −0.4)	.03	−4.0 (−9.3 to 1.6)	.11	−4.8 (−7.9 to −1.6)	.02	−4.4 (−9.1 to 0.4)	.06	−4.3 (−11.4 to 3.3)	.17	−4.0 (−7.9 to 0.1)	.05
65-69	−5.1 (−9.6 to −0.4)	.04	−4.3 (−8.3 to −0.3)	.04	−3.6 (−9.3 to 2.5)	.16	−2.3 (−10.9 to 7.1)	.48	−3.1 (−8.2 to 2.3)	.16	−3.7 (−9.0 to 1.9)	.12
70-74	−2.7 (−7.1 to 1.9)	.16	−2.8 (−8.4 to 3.1)	.22	−2.5 (−8.9 to 4.3)	.31	−1.2 (−8.1 to 6.3)	.64	−1.1 (−7.2 to 5.3)	.61	−2.1 (−7.9 to 4.0)	.34
75-79	−4.7 (−7.6 to −1.6)	.02	−3.5 (−7.4 to 0.6)	.07	−2.7 (−9.4 to 4.5)	.31	−2.6 (−6.5 to 1.5)	.14	−3.3 (−11.0 to 5.0)	.29	−3.4 (−8.3 to 1.8)	.13
80-84	−4.6 (−8.1 to −0.8)	.03	−2.8 (−6.2 to 0.8)	.09	−1.6 (−3.5 to 0.4)	.09	1.1 (−4.8 to 7.3)	.61	−1.0 (−5.9 to 4.0)	.55	−2.0 (−5.0 to 1.1)	.13
85-89	−2.8 (−8.8 to 3.7)	.26	−1.6 (−9.6 to 7.1)	.58	0.2 (−6.9 to 8.0)	.93	0.8 (−5.4 to 7.4)	.72	−0.8 (−11.0 to 10.6)	.83	−1.0 (−7.6 to 6.2)	.69
≥90	−2.4 (−10.8 to 6.7)	.45	−1.7 (−11.9 to 9.7)	.66	1.9 (−8.5 to 13.4)	.62	1.4 (−10.2 to 14.6)	.74	−0.8 (−13.2 to 13.4)	.86	−0.5 (−9.9 to 9.8)	.88

Abbreviation: APC, average percent change.

^a^ Q1 income level represents the lowest income quintile; Q5 represents the highest income quintile.

Colorectal cancer incidence rates were also highest for Q4 and Q5 for individuals aged 50 to 54 years and increased, but nonsignificantly, for Q1 from 8.73 per 100 000 (95% CI, 7.95-9.51) in 1992-1996 to 9.89 per 100 000 (95% CI, 9.29-10.49) in 2012-2016. Colorectal cancer incidence rates were fairly stable for all other income quintiles. Unlike the rates in younger age groups, CRC incidence rates decreased for individuals aged 55 to 74 years in all income quintiles. For those aged 55 to 59 years, the rates were highest for Q5 from 1992-1996 (17.97 per 100 000; 95% CI, 16.76-19.18) until 2012-2016, when incidence rates became lower for Q5 than for other income quintiles (14.56 per 100 000; 95% CI, 13.80 to 15.32). Colorectal cancer incidence rates decreased significantly for Q2 (APC, −4.1%, 95% CI, −6.7% to −1.5%). Colorectal cancer incidence rates were similar by income quintile among those aged 60 to 64, 65 to 69, and 70 to 74 years. The decrease was significant for individuals aged 60 to 64 years in Q1 (APC, −2.3%; 95% CI, −4.2% to −0.4%) and Q3 (APC, −4.8%; 95% CI, −7.9% to −1.6%) and for individuals aged 65 to 69 years in Q1 (APC, −5.1%; 95% CI, −9.6% to −0.4%) and Q2 (APC, −4.3%; 95% CI, −8.3% to −0.3%). No decrease was significant for individuals aged 70 to 74 years. Colorectal cancer incidence rates also decreased for individuals aged 75 to 79 years from 66.43 per 100 000 (95% CI, 65.00-67.87) in 1992-1996 to 57.34 (95% CI, 56.24-58.45) in 2012-2016. Colorectal cancer incidence rates were highest for Q1 and Q2 and lowest for Q5. The decrease was significant for Q1 (APC, −4.7%; 95% CI, −7.6% to −1.6%). For individuals aged 80 to 84, 85 to 89, and 90 or more years, CRC incidence rates were highest for Q1 and Q2. The incidence decreased significantly over time only for those aged 80 to 84 years in Q1 (APC, −4.6%; 95% CI, −8.1% to −0.8%) and remained fairly stable for Q4 and Q5.

**Figure 2.**
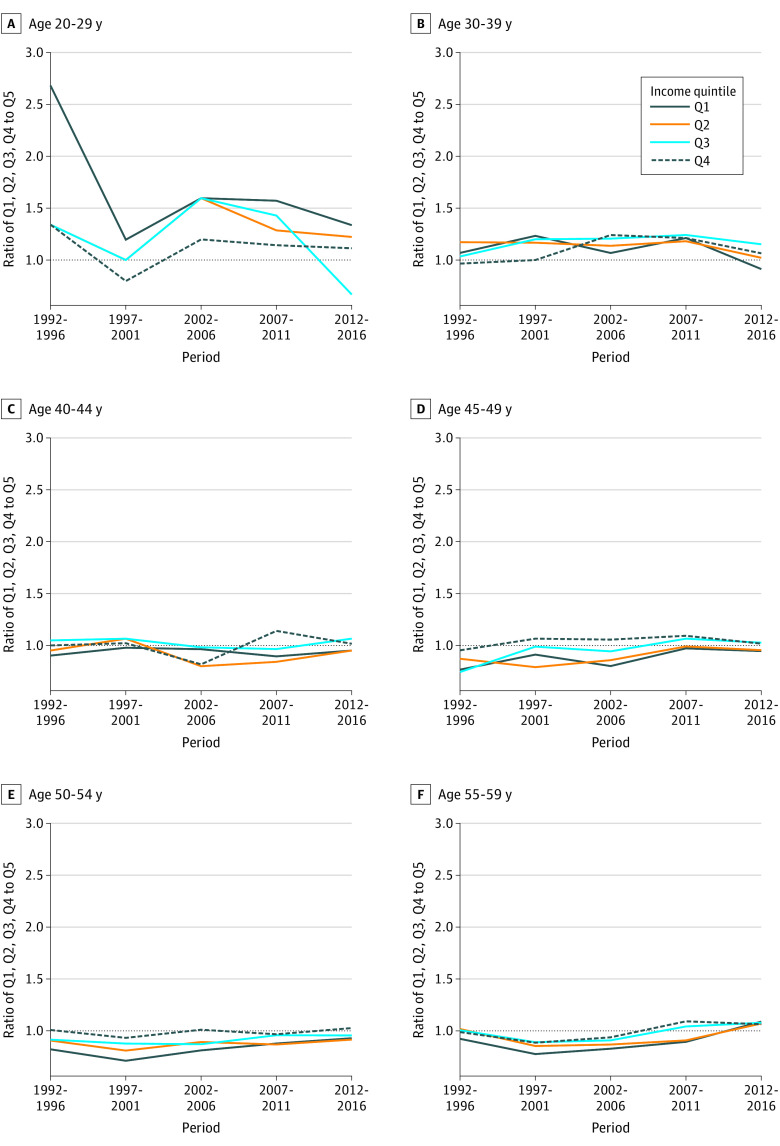
Ratio of Colorectal Cancer Incidence Rate by Income Quintile and Age Group for Both Sexes Combined in Canada by 5-Year Time Periods From 1992 to 2016

Figure 2 and eFigure 2 in the Supplement compare CRC incidence rates for income quintiles Q1 to Q4 with the highest income quintile (Q5) by age group for both sexes combined in Canada. Individuals aged 20 to 29 and 30 to 39 years in Q1 and Q2 had higher CRC incidence rates compared with individuals in Q5. The ratio between the maximum and minimum CRC incidence rates was highest among the 20- to 29-year age group from 1992 to 1996 (ratio, 2.67; 95% CI, 1.47-4.83) across all income groups and 2012 to 2016 (ratio, 2.00; 95% CI, 1.29-3.10) across all income groups (Table 3). This time trend changed for the 40- to 44-year and 45- to 49-year age groups when lower income quintiles had lower CRC incidence rates than the higher income quintiles, and variability between income quintiles decreased. A similar pattern was seen for individuals aged 50 to 54 and 55 to 59 years. In the 65- to 69-year group, CRC incidence rates started to increase again for the lower income groups relative to Q5 but with little variability between income quintiles. There was more variability by income quintile for individuals aged 70 to 74 and 75 to 79 years, although CRC incidence rates remained higher for Q1 and Q2. Variability by income quintile increased for the oldest age groups (80-84, 85-89, and ≥90 years), but CRC incidence rates remained highest for the lowest income groups.

**Table 3.** Ratio of Colorectal Cancer Incidence Rates Between the Maximum and Minimum Income Quintile Within Each Age Group

Age, y	1992-1996		1997-2001		2002-2006		2007-2011		2012-2016	
	Ratio (95% CI)	*P* value	Ratio (95% CI)	*P* value	Ratio (95% CI)	*P* value	Ratio (95% CI)	*P* value	Ratio (95% CI)	*P* value
20-29	2.67 (1.47-4.83)	.001	1.50 (0.85-2.64)	.16	1.60 (0.97-2.64)	.07	1.57 (1.03-2.40)	.04	2.00 (1.29-3.10)	.002
30-39	1.21 (0.97-1.52)	.09	1.23 (0.99-1.53)	.06	1.24 (1.00-1.54)	.05	1.24 (1.01-1.53)	.04	1.26 (1.05-1.51)	.01
40-44	1.16 (0.95-1.41)	.13	1.09 (0.91-1.30)	.36	1.25 (1.05-1.49)	.01	1.35 (1.15-1.60)	<.001	1.12 (0.96-1.31)	.16
45-49	1.34 (1.16-1.55)	<.001	1.35 (1.18-1.54)	<.001	1.32 (1.16-1.49)	<.001	1.12 (1.00-1.27)	.05	1.09 (0.96-1.22)	.17
50-54	1.23 (1.09-1.38)	.001	1.41 (1.26-1.56)	<.001	1.25 (1.13-1.37)	<.001	1.15 (1.05-1.26)	.002	1.12 (1.03-1.22)	.008
55-59	1.10 (1.00-1.21)	.05	1.29 (1.18-1.41)	<.001	1.21 (1.12-1.31)	<.001	1.22 (1.13-1.32)	<.001	1.09 (1.01-1.17)	.02
60-64	1.07 (0.99-1.16)	.09	1.13 (1.05-1.23)	.002	1.09 (1.02-1.18)	.02	1.12 (1.05-1.20)	.001	1.09 (1.02-1.16)	.01
65-69	1.16 (1.08-1.25)	<.001	1.11 (1.04-1.19)	.002	1.12 (1.04-1.19)	.001	1.11 (1.05-1.19)	.001	1.10 (1.04-1.17)	.001
70-74	1.21 (1.13-1.29)	<.001	1.15 (1.08-1.23)	<.001	1.17 (1.10-1.24)	<.001	1.13 (1.06-1.19)	<.001	1.12 (1.06-1.19)	<.001
75-79	1.27 (1.18-1.36)	<.001	1.22 (1.15-1.30)	<.001	1.20 (1.13-1.28)	<.001	1.18 (1.12-1.26)	<.001	1.28 (1.20-1.36)	<.001
80-84	1.43 (1.33-1.55)	<.001	1.37 (1.27-1.47)	<.001	1.27 (1.19-1.36)	<.001	1.19 (1.11-1.26)	<.001	1.26 (1.18-1.34)	<.001
85-89	1.38 (1.24-1.54)	<.001	1.40 (1.27-1.54)	<.001	1.35 (1.23-1.47)	<.001	1.21 (1.12-1.31)	<.001	1.39 (1.29-1.50)	<.001
≥90	1.56 (1.31-1.85)	<.001	1.66 (1.43-1.93)	<.001	1.45 (1.28-1.66)	<.001	1.33 (1.18-1.50)	<.001	1.50 (1.34-1.68)	<.001

## Discussion

### Main Findings

In this study, we examined time trends in CRC incidence rates by age group and income quintile for Canada from 1992 to 2016 using national data from the Canadian Cancer Registry and Statistics Canada. To our knowledge, the use of smaller age groups and the association of CRC incidence rates over time with income quintile have not been previously examined for Canada. We found that CRC incidence rates for Canadians aged 20 to 44 years increased for all income quintiles, with higher incidence rates in the lower income quintiles. For individuals aged 45 to 49 years, CRC incidence rates increased significantly for individuals in the lowest income quintile and, for those aged 50 to 54 years, CRC incidence rates were stable for all income quintiles. For both age groups, CRC incidence rates were higher for the higher quintiles, and variability between income quintiles was low. For individuals aged 55 to 74 years, CRC incidence rates were stable or decreased for all income quintiles; incidence rates were also higher for the highest income quintiles. There was less variability in CRC incidence rates by income quintile particularly by 2012-2016. In individuals older than 75 years, CRC incidence rates were stable or decreasing, became highest for the lowest income quintiles, and variability between income quintiles increased.

Although a small percentage of the total number of CRC cases in Canada occurs among individuals aged 20 to 29 and 30 to 39 years, the increasing incidence rates and high variability between income quintiles are concerning. The association between low income and increased health risks is well established.^12^ The increase in CRC incidence rates among younger individuals in Canada with lower incomes may be partially explained by an increase in CRC risk factors, such as decreased rates of physical activity, lower fruit and vegetable intake, and higher obesity rates.^12,16^ This issue must be addressed since younger adults with cancer already face significant challenges, including delays in diagnosis, because cancer is uncommon and awareness and suspicion of cancer are low in this population.^17^ The increasing or stable CRC incidence rates among individuals aged 45 to 50 and 50 to 54 years are likely owing to lower screening rates in these age groups compared with older age groups.^18^

In contrast, the reduction in CRC incidence rates in individuals aged 55 to 74 years is likely related to the introduction of CRC screening. In 2001, the National Committee of Health Canada recommended screening for CRC using a fecal test every 2 years for individuals aged 50 years or older.^19^ This recommendation led to the implementation of population-based organized CRC screening programs in most Canadian provinces beginning in 2007. The greater variability in CRC incidence rates by income quintile outside of the age group targeted for screening (50-74 years) in Canada suggests that organized screening in a universal health care setting can reduce disparities in CRC incidence. Similarly, the increased variability in CRC incidence rates by income quintile after age 75 years likely reflects the fact that many older individuals would not have been eligible for organized screening.

### Comparison With Other Studies

Previous analyses of CRC incidence rates over time in Canada found increases in incidence among men and women younger than 50 years and 40 to 49 years.^6,7,8^ Our results suggest that the increase in CRC incidence rates is occurring in the 20- to 45-year age range and less so among individuals aged 45 to 50 years. Hence, reducing the start age of CRC screening to 45 years in Canada, as has been recommended by the American Cancer Society, may likely have limited effect.^20^

The association between SES and CRC incidence rates appears to vary by geography. A systematic review of 21 cross-sectional studies that examined the association between CRC incidence rates and SES (measured using income, educational level, and various other SES indices) found an association between increased risk of CRC and low SES in the US; however, the risk was reduced or nonsignificantly altered in European studies.^16^ Another review that included 19 studies also suggested that lower SES was generally associated with a higher risk of CRC in the US and Canada but a lower risk in Europe.^21^ Studies from Australia and South Korea reported lower CRC incidence rates among individuals with a low SES.^21^ The variabilities between countries are likely owing to differences in diet, screening participation, and perhaps access to health care.

Fewer studies have examined the association between CRC incidence rates and SES over time. Liang et al^22^ examined temporal trends in sociodemographic disparities in CRC among Medicare patients in the US from 1973 to 2010. Similar to the results from our study, they found that higher SES (educational level and income quintile) was associated with a greater increase in CRC incidence rates from 1973 to 1997 but became protective after 1998. Teng et al^23^ examined CRC incidence and mortality from 1985 to 2011 in New Zealand. Although they found a crossover in CRC mortality rates from being highest in high SES groups to highest in low SES groups, there were no significant trends in absolute inequalities in CRC incidence rates for either men or women.

Income inequalities in CRC incidence rates appear to be emerging in the US. Zhang et al^24^ investigated the association between changes in neighborhood SES and CRC incidence rates among individuals aged 50 to 71 years who lived in the same location for at least 10 years in several US states. Compared with individuals living in neighborhoods with long-term high SES, those living in neighborhoods with consistently low SES or in neighborhoods that experienced a decrease in SES over 10 years had a higher risk of CRC. In addition, Liu et al^25^ examined whether median household income was associated with CRC incidence among individuals in Texas from 1995 to 2011. They reported that CRC incidence rates slightly decreased with increasing median income and the gap in CRC incidence rates between high and low SES was narrowing. However, to our knowledge, there are no prior data on variation in CRC incidence rates in the younger population by SES in the US.

### Strengths and Limitations

The results of this study should be interpreted in the context of its strengths and limitations. We used data from the Canadian Cancer Registry, the population-based database that collects information on cancer incidence from all provincial and territorial cancer registries in Canada since 1992. The quality of the data is continuously monitored to ensure that the information in the Canadian Cancer Registry^26^ meets standards for acceptance in international publications, such as *Cancer Incidence in Five Continents*^27^ and *Cancer Incidence in North America*.^28^ The percentage of cases that were missing a postal code at diagnosis was low. We were able to use area-level income quintile as a measure of SES. Prior studies have reported a correlation between a neighborhood-level and a self-reported income.^29,30^ In addition, a neighborhood-level measure of income takes into account the socioeconomic context of the individuals who reside in the same dissemination area.

Limitations of the study include the lack of information on individuals from Québec, which was not included because data were available only until 2010. In addition, we did not examine trends in CRC incidence rates among different age groups by sex, and the small number of cases in the younger age groups could lead to limited power in comparisons and unstable rates.

## Conclusions

This study found that trends in CRC incidence rates differed by age group and income quintile. Among Canadians aged 20 to 49 years, CRC incidence rates increased for all income quintiles, with higher incidence rates in the lower income quintiles. For individuals for whom CRC screening is recommended (ages 50-74 years), CRC incidence rates decreased for all income quintiles, with little variability between income quintiles. In individuals older than 75 years, CRC incidence rates were stable or decreased, with increased higher incidence among the lower income quintiles. These results suggest that, although population-based screening can reduce income disparities, targeted interventions and further research are needed to address the increasing CRC incidence rates among younger individuals in Canada, particularly in the lower income quintiles.

## Notes

Supplement. **eFigure 1.** Time Trends in Colorectal Cancer Incidence Rate per 100,000 Individuals by Income Quintile and Age Group for Both Sexes Combined in Canada by Five-Year Time Periods From 1992 to 2016 **eFigure 2.** Ratio of Colorectal Cancer Incidence Rate by Income Quintile and Age Group for Both Sexes Combined in Canada by Five-Year Time Periods From 1992 to 2016
