# Supplementary material for: Time Trends in Colorectal Cancer Incidence Rates by Income and Age at Diagnosis in Canada From 1992 to 2016
Source: JAMA Netw Open. 2021 Jul 19;4(7):e2117556. doi: 10.1001/jamanetworkopen.2021.17556 (PMC8290311; doi:10.1001/jamanetworkopen.2021.17556)
Supplement: Supplement. — eFigure 1. Time Trends in Colorectal Cancer Incidence Rate per 100,000 Individuals by Income Quintile and Age Group for Both Sexes Combined in Canada by Five-Year Time Periods From 1992 to 2016 eFigure 2. Ratio of Colorectal Cancer Incidence Rate by Income Quintile and Age Group for Both Sexes Combined in Canada by Five-Year Time Periods From 1992 to 2016 [file jamanetwopen-e2117556-s001.pdf]

Supplementary Online Content

Decker KM, Lambert P, Bravo J, Demers A, Singh H. Time trends in colorectal cancer incidence rates by income and age at diagnosis in Canada from 1992 to 2016. *JAMA Netw Open*. 2021;4(7):e2117556. doi:10.1001/jamanetworkopen.2021.17556

eFigure 1. Time Trends in Colorectal Cancer Incidence Rate per 100,000 Individuals by Income Quintile and Age Group for Both Sexes Combined in Canada by Five-Year Time Periods From 1992 to 2016

eFigure 2. Ratio of Colorectal Cancer Incidence Rate by Income Quintile and Age Group for Both Sexes Combined in Canada by Five-Year Time Periods From 1992 to 2016

This supplementary material has been provided by the authors to give readers additional information about their work.

© 2021 Decker KM et al. *JAMA Network Open.*

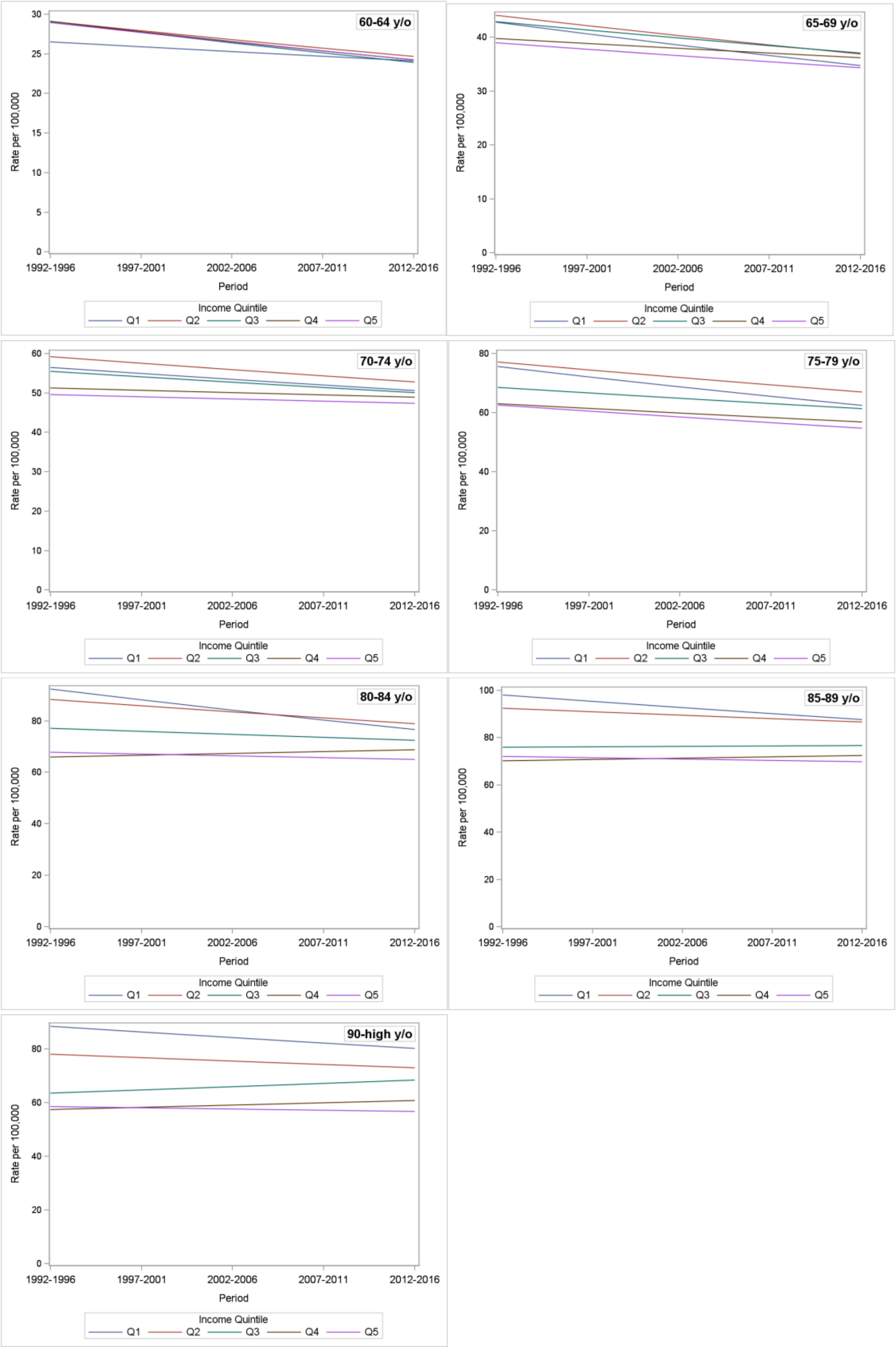

eFigure 1: Time trends in colorectal cancer incidence rate per 100,000 individuals by income quintile and age group for both sexes combined in Canada by five-year time periods from 1992 to 2016

© 2021 Decker KM et al. *JAMA Network Open.*

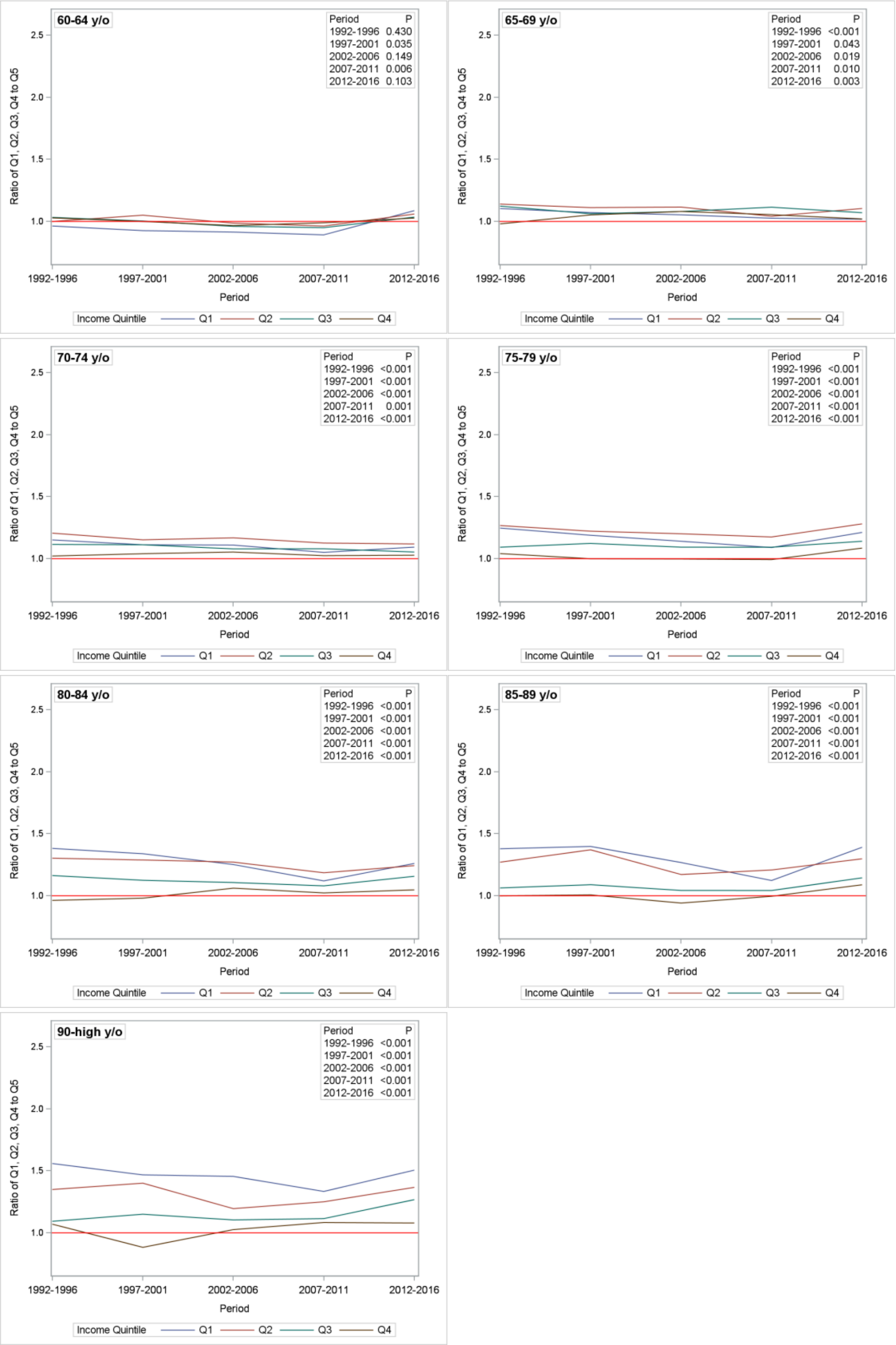

eFigure 2: Ratio of colorectal cancer incidence rate by income quintile and age group for both sexes combined in Canada by five-year time periods from 1992 to 2016

© 2021 Decker KM et al. *JAMA Network Open.*
